# Bioinformatic and empirical analysis of a gene encoding serine/threonine protein kinase regulated in response to chemical and biological fertilizers in two maize (*Zea mays* L.) cultivars

**Published:** 2017-06

**Authors:** Ida Azad, Abbas Alemzadeh

**Affiliations:** 1 Department of Crop Production and Plant Breeding, School of Agriculture, Shiraz University, Shiraz, Iran; 2 Plant Breeding and Biotechnology Department, Agricultural Sciences and Natural Resources University of Sari, Sari, Iran

**Keywords:** Gene expression regulation, Phosphate starvation stress, Plant protein kinase, *ZmSTPK1*

## Abstract

Molecular structure of a gene, *ZmSTPK1*, encoding a serine/threonine protein kinase in maize was analyzed by bioinformatic tool and its expression pattern was studied under chemical biological fertilizers. Bioinformatic analysis cleared that *ZmSTPK1* is located on chromosome 10, from position 141015332 to 141017582. The full genomic sequence of the gene is 2251 bp in length and includes 2 exons. Its cDNA length is 1900 bp with a 5'-untranslated region of 311 bp and 3'-untranslated region of 341 bp, of which 1248 bp from open reading frame encoding 415 amino acid residues with a molecular weight of 46 kDa and an isoelectric point 7.2. Also, an upstream open reading frame contains 100 aa was found at -12 position from ATG initiation codon. ZmSTPK1 with a long half-life, 10 hours in *Escherichia coli*, and instability index of 32.25 is classified as a stable protein. A calmodulin binding domain was found in ZmSTPK1 at position from 395 to 405 in C-terminal end. The helical wheel analysis showed that the stretch of residues Ile-395 to Asp-415 has the potential to form a charged amphiphilic -helix characteristic of a calmodulin-binding region. Two *P1BS*-like motifs, which are present in the promoter regions of Pi starvation-induced genes, were located at positions -48 and -867 from ATG initiation codon. The expression of *ZmSTPK1* responded to available phosphate, and its expression up-regulated under phosphate starvation.

## Introduction

Maize is one of the most widely grown crop species in the world and represents an important source of food, feed, biofuel, and industrial products [1]. Soils contain natural reserves of plant nutrients, but these reserves are mainly in forms unavailable to plants, and only a small part is released each year through chemical processes or biological activity. Soil microorganisms play an important role in regulating the dynamics of organic matter decomposition and the availability of plant nutrients such as N, P and K. [2]. As an essential element for all plant cells, phosphorus is indispensable nutrient in all agricultural production systems, but natural phosphorus reserves are limited [3]. 

Some bacteria and fungi can make insoluble phosphorus available to the plant [3]. In different plant species, the interaction between plant and microorganism has analyzed at the cellular and molecular levels. Genes that were differentially regulated were involved in primary and secondary metabolisms, response to stimuli, cell organization, protein modification and transcriptional regulation [2, 4, 5]. 

In nature, plants are frequently affected by the surrounding conditions and their response to these conditions, including changes in the expression of various genes, have been reported [6, 7]. Protein phosphorylation is one of the most versatile posttranslational modifications used in eukaryotic cells in response to environmental changes and plays an important role in the continuous remodeling of different transcriptional regulators [8]. Protein kinases are a class of enzymes which use ATP to phosphorylate other proteins within the cell and hereby play an important role in the control of many aspects of cellular life [9, 10]. They can be divided into three major subclasses including receptor tyrosine kinases (RTK), serine/threonine protein kinases (STPK) and histidine kinases [11, 12]. It has been reported that after inoculation, various genes induced due to interaction between plant and microorganism [13]. Various studies showed that the transcriptional responses occurred in plants during development of fungal or bacterial symbiosis [14, 15]. It also showed that some induced genes are required for the formation of symbiosis between plants and bacteria [16]. Interestingly, the studies of individual genes have revealed that some fungal- or bacterial-regulated genes are constitutively expressed in plants and up-regulated in symbiosis, while others are expressed only during fungal or bacterial symbiosis [2, 17]. In addition, it has shown that a subset of these genes is regulated as a consequence of alternations in the phosphate status of the plant [2]. In rice, around 40% of the fungal-regulated genes were differentially expressed in response to the interaction with one of two different fungi, indicating an overlap in the responses to these fungi [14, 18]. In *Medicago truncatula*, more overlap in the transcriptional responses to different fungi has been observed [19]. 

There are various references in the literature that address the expression of kinase genes changed in response to different environmental conditions [20], but there is no report, to our knowledge, to show the expression of kinase genes are regulated by chemical and biofertilizers. Hence, in this work we have used maize as a crop plant to study the effect of biofertilizers and chemical phosphate fertilizers on the expression pattern of a gene encoding serine/threonine protein kinase, because the characterization of environmental-induced genes have greatly contributed to our understanding of the physiological responses of plant cells at the molecular level to different environmental factors. We also tried to analyze the structure of this protein and its encoding gene to reveal important motifs and *cis*-elements associated with its function.

## MATERIALS AND METHODS

**Cultivation of plants and experimental design: **A green house experiment was conducted to assess the effect of Barvar2, as phosphate biofertilizer, and triple super phosphate, as chemical fertilizer, on the expression of a gene encoding serine/threonine protein kinase in two maize cultivars, Hido and 677. For this purpose, a two factorial experiment, laid out in a randomized complete design with three replications. The factors included fertilizer at 4 levels (triple super phosphate, 200 kg/ha (chemical A) and 100 kg/ha (chemical B), 100 g/ha Barvar2 and control (without fertilizer)), and two cultivars, Hido and 677. 

The seeds of maize cultivars were obtained from Seed and Plant Improvement Institute of Iran. The obtained seeds were sterilized by soaking in 2.5% sodium hypochlorite for 5 minutes and then washed three times with distilled water. Afterwards, the sterile seeds were planted in plastic pots filled with sand and grit (1:1 v/v) in a controlled-environment growth room under 8/16 h light/dark photoperiod at 16-25°C [21]. All fertilizer treatments were applied at planting time. For biofertilizer, the seeds were inoculated with barvar2 before planting. Plants were regularly irrigated with tap water and two weeks after emergence, the plants were thinned to three plants per pot. In the early stages of growth, from 4 to 6 leaves, leaf samples were harvested and immediately frozen in liquid nitrogen, and stored at -80ºC until used.

**Primer design: **cDNA sequence of a gene encoding a serine/threonine protein kinase in maize, *ZmSTPK1* gene, was obtained from GenBank (accession No. EU964756). Two specific primers (STPKF: 5'- AAC ATG ACA GCA GTG CAA GC- 3'; STPKR: 5'-CTG AGG TGG AGA TCC TGA GC -3') were designed by AlleleID software to amplify a 186 bp fragment from coding sequence region of *ZmSTPK1*. Also, two specific primers (ZmUBQ2F: 5'- CTT TGC TGC TGC ACG GGA GGA ATG -3'; ZmUBQ2R: ATG GAC GCA CGC TGG CTG ACT A -3') designed by Deng et al. (2014) used to amplify a 177 bp fragment from 3'-untranslated region of Zm*UBQ2* gene, a gene encoding ubiquitin in maize, as reference gene.

**DNA extraction and purification:** Frozen leaves were used for DNA extraction. DNA was extracted by cetyltrimethylammonium bromide,CTAB method [23] with some modifications [24]. The DNA extraction buffer contained 143 mL of deionized water; 22 mL of Tris 1M, pH 7.5; 22 mL EDTA 0.5M, pH 8 and 30.8 mL NaCl 5M. The leaves were grinded with a mortar and pestle in liquid nitrogen as finely as possible. Around 400 mg of powdered seeds were transferred to a 50 mL sterile tube followed by addition of 9 mL hot extraction buffer and incubated at 65°C for 60 minutes with occasional inverting of the tubes. The tube was left at room temperature for 10 minutes without shaking. Afterward, 4.5 mL octanol:chloroform (24:1, v/v) was added to each tube, mixed thoroughly and centrifuged at 1420 g for 15 minutes. The supernatant was transferred to a new tube and 4.5 mL octanol:chloroform (24:1, v/v) was added to each tube. Tubes were shaken for 10 minutes at room temperature. The tubes were centrifuged at 1420 g for 15 minutes. The supernatant was transferred to a new tube, 10 µL RNase was added to each tube and was left at room temperature for one hour. After that, 12 mL cold absolute ethanol was added to each tube and centrifuged at 1420 g for 10 minutes at room temperature. After centrifugation the supernatant removed and DNA was resuspended in 100 µL of sterile deionized water and stored at -20°C until used.

For purification, DNA was precipitated with absolute ethanol and washed by solution I (absolute ethanol, 76 mL; acetate sodium 1 M, 8 mL; deionized water, 16 mL) and solution II (absolute ethanol, 76 ml; acetate ammonium 1 M, 1 ml; deionized water, 23 mL), respectively. The quantity and quality of DNA was assessed by electrophoresis on 1% garose gel. 

**RNA extraction and preparing cDNA:** Frozen leaves were used for RNA extraction. Total RNA were extracted by the GF-1 Total RNA Extraction Kit and treated with DNase I [25]. The quality of the extracted RNA was assessed by electrophoresis on 1% agarose gel [26]. cDNA synthesis was performed by RevertAid M-MuLV reverse transcriptase and oligo (dt) primer using 1 µg of total RNA according to the manufacturer's instructions. The concentrations of all prepared cDNAs were determined by Nanodrop device. 

**PCR reaction:** The cDNA was amplified by specific primers for *ZmSTPK1* and *UBQ2* genes, as target and reference genes, respectively. PCR reactions were carried out in a final volume of 25 μl reaction mixture containing 10 mM tris-HCl (pH 8.3), 50 mM KCl, 1.5 mM MgCl_2_, 200 µM dNTPs, 1 µl diluted cDNA, 0.3 µM of each primer and 1 unit taq DNA polymerase were performed under the following conditions: 3 min at 94°C, followed by 29 cycles one minute at 95°C, 30 s at 57°C for *ZmSTPK1* gene or 55°C for *UBQ2* gene, and 1 min at 72°C, with a final extension step of 15 min at 72°C. The PCR products were then separated on 1% agarose gel.

**Semi-** **quantitative** **analysis** **of gene expression: **After electrophoresis, the gel was photographed by UV transillumination, the intensity of bands quantified by Total-Lab software.

**Bioinformatic analysis** **:** The position of gene on the chromosomes of maize was determined by Eukaryotic Genome Annotaion tool, NCBI http://www.ncbi.nlm.nih.gov/ genome/annotation_euk/all/). The various physical and chemical parameters included estimated half-life, instability index and amino acid composition of protein, were determined by ProtParam tool, ExPASy (http://web.expasy.org/protparam/). The nucleotide sequences were translated to amino acid sequences using translate tool on the SIB Bioinformatics Resource Porta, ExPASy (http://web.expasy.org/translate/). The molecular weight was computed by compute PI/MW tool, ExPASy (http://web.expasy. org/compute_pi/). Upstream *cis*-acting regulatory element sequences of *ZmSTPK1* were analyzed by PlantCARE program (http://bioinformatics.psb.ugent.be/webtools/ plantcare/html/) [27]. The helical wheel representation was drawn using the Heliquest software (http://heliquest.ipmc.cnrs.fr/cgi-bin/ComputParamsV2.py).

## RESULTS AND DISCUSSION

**Figure 1:**
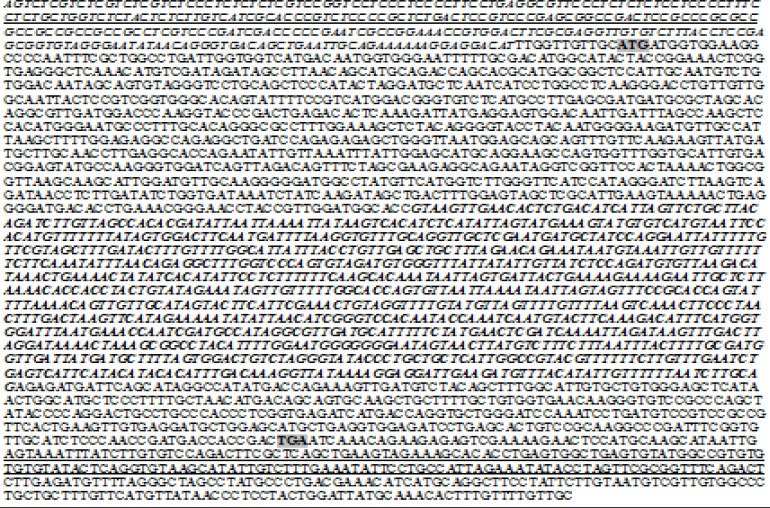
The nucleotide sequence of *ZmSTPK1* gene from *Zea mays.* The gene is 2251 bp in length which expresses a transcript with 1248 bp in length. Start and stop codons are shown in bold and gray and intron region (1003 bp) is shown in bold and italic. 5'-UTR (311 bp) and 3'-UTR (341 bp) are underlined. An upstream open reading frame (uORF) (89 aa), MSSFFLQFSCHPVIFPTPLGGKDTTSRSPRFPAIRG SIGTRRRRRRRGRSRPLGTESERGDGCDDKRVETSRGKGRRERERLRKGREDRTRERETRRDET, in upstream of *ZmSTPK1* at -12 position from ATG initiation codon is shown in italic and underlined

The sequence of *ZmSTPK1* was analyzed using bioinformatic tools. The results cleared that the maize *ZmSTPK1* gene is located on chromosome 10, from position 141015332 to 141017582. The gene was 2251 bp in length with an ORF of 1248 bp encoding 415 amino acid residues and a 5'-UTR of 311 bp and 3'-UTR of 341 bp (Fig. 1). The comparison of the genomic DNA and cDNA sequences (GeneBank accession No. EU964756.1) revealed that this gene includes only one intron (1003 bp) which separates two exons (Fig. 1). The sequences of the intron/exon splice junctions follow the GT-AG rule [28].

Also, an upstream open reading frame (uORF) contains 100 aa, MSSFFLQFSCHPV IFPTPLGGKDTTSRSPRFPAIRGSIGTRRRRRRRGRSRPLGTESERGDGCDDKRVETSRGKGRRERERLRKGREDRTRERETRRDET, was found at- 12 position from ATG initiation codon (Fig. 1). This uORF was also reported for other genes like H^+^-ATPases and Na^+^/H^+^ antiporter and supposed to regulate the translation of a downstream gene [29, 30]. 

The predicted molecular weight of ZmSTPK1 is 46 kDa with an isoelectric point (PI) 7.2. Its PI is in the range of plant cell cytosol pH [31], it appears that ZmSTPK1 has close-to-optimal activity in the cytosol compartments. Interestingly, the percentage of negatively and positively charged residues is equal in ZmSTPK1, which may cause its PI to be 7.2. In addition, instability index was computed to be 32.25, which classifies the protein as stable one. When the half-life of ZmSTPK1 was estimated, it was calculated 10 hours in *Escherichia coli*, which confirms its stability. The result is consistent with those of other studies that showed kinase proteins are stable proteins with a long half-life [32].

**Figure 2:**
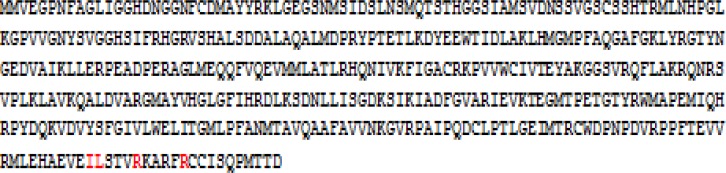
Deduced amino acid sequences of ZmSTPK1. The IQ motif, **IL**STV**R**KARF**R**, was found at position from 395 to 405 in C-terminal end

Calmodulin is recognized as an important calcium sensor in plant cells regulators a various group cellular proteins through binding with these proteins [33αwhere x represents any amino acid [33]. IQ motif located in a stretch of 17-25 amino acid residues having a charged amphiphilic region and a tendency to form an α^34^]. The IQ motif, **IL**STV**R**KARF**R**, was found in ZmSTPK1 at position from 395 to 405 in C-terminal end (Fig. 2).

This motif was also reported from other regulatory proteins [35]. αFig. 3). In the sequence of IQ motif in ZmSTPK1, Q (Gln) replaced with L (Leu) that may be the result of a point mutation. In the sequence of *ZmSTPK1*, the nucleotide at position 1187 is “T” that converted the codon of Gln (C**A**G) to Leu (C**T**G). An empirical study is necessary to determine the effect of this mutation. Around 1500 bp upstream from the start codon was determined and analyzed as promoter region. Most abundant motif was *cis*-acting element involved in light responsiveness although, there were various *cis*-acting elements in the upstream region of *ZmSTPK1* (Table 1). It was also some elements involved in the abscisic acid responsive, some plant hormones like methyl jasmonate or auxin -responsiveness and MYB binding site involved in drought-inducibility. The results indicated that this gene must be involved in abiotic stress such as phosphate starvation stress.

**Figure 3:**
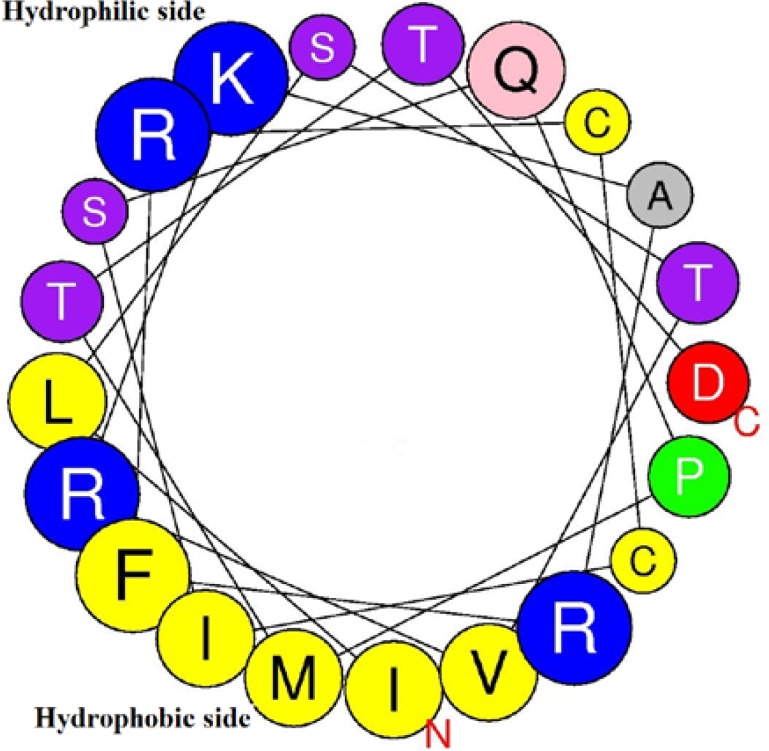
Helical-wheel model of 435-to-455 aa region of ZmSTPK1. Hydrophilic and hydrophobic sides are shown

Phosphorus is an essential nutrient for plant growth and development, and is used as the most common fertilizer component after nitrogen. Hence, we used 1500 bp sequence upstream of the start codon of *ZmSTPK1* gene to search for the motifs involved in phosphate starvation signaling. The analysis showed that two *P1BS*-like sequences, which is present in the promoter regions of Pi starvation-induced genes, were located at positions -48 and -867 from ATG initiation codon. *P1BS* is one of the most important elements (GNATATNC) in phosphate starvation signaling was reported by Rubio et al. (2001). It has been previously reported that the existence of *P1BS*-like motif is required for the regulation of phosphate starvation signal through some transcription factors such as OsPHR2 [37]. Bustos et al. (2010) showed that *P1BS* is conserved in phosphate starvation-responsive genes and plays an important role in phosphate starvation as an integrating *cis* regulatory element associated with genes that are induced by phosphate starvation. Hence, we performed a transcriptional analysis of *ZmSTPK1* gene under different phosphate.

The quality of extracted DNA and RNA was assessed by electrophoresis on agarose gel. When the total RNA sample was subjected to electrophoresis, bands corresponding to 28S and 18S rRNA were distinctly visible, indicating high quality and non-degraded RNA. When genomic DNA was subjected to electrophoresis, a distinct and strong band with no smear appeared, indicating intact and non-degraded DNA.

RT-PCR is a sensitive method to degradation of RNA or the presence of inhibitors in the extract [26]. Hence, RT followed by a standard PCR may be used to test RNA quality and the quality of cDNA synthesis. After extraction of total RNA from leaves, cDNA was prepared and subjected to a standard PCR with specific primers for *ZmSTPK1* and *ZmUBQ2* genes (described in Materials and Methods) to amplify fragments from these genes. A 186 bp fragment from *ZmSTPK1* cDNA and a 177 bp fragment from *ZmUBQ2* cDNA were successfully amplified that indicate the high-quality of extracted RNA and prepared cDNA.

**Table 1:** Identification *cis* elements in the upstream region of *ZmSTPK*

**Motif**	**Number of motif**	**Function of motif**
A-box	6	*cis*-acting regulatory element associated with P-and L-box involved in induced transcriptional activity
ABRE	11	*cis*-acting element involved in the abscisic acid responsive
ACE	6	*cis*-acting element involved in light responsiveness
ARE	4	*cis*-acting regulatory element essential for the anaerobic induction
BOX 4	1	part of a conserved DNA module involved in light responsiveness
CAAT-box	9	common *cis*-acting element in promoter and enhancer regions
CAAT-motif	1	part of a light responsive element
CCAAT-box	2	MYBHv1 binding site
CGTCA-motif	1	cis-acting regulatory element involved in the MeJA (methyl jasmonate)-responsiveness
G-Box	6	*cis*-acting regulatory element involved in light responsiveness
G-box	12	*cis*-acting regulatory element involved in light responsiveness
GAG-motif	1	part of a light responsive element
GC-motif	9	enhancer-like element involved in anoxic specific inducibility
GCN4-motif	4	*cis*-regulatory element involved in endosperm expression
MBS	1	MYB binding site involved in drought-inducibility
O_2_-site	5	*cis*-acting regulatory element involved in zein metabolism regulation
Skin-1-motif	1	*cis*-acting regulatory element required for endosperm expression
Sp1	1	light responsive element
TATA-box	26	core promoter element around -30 of transcription start
TCCC-motif	2	part of a light responsive element
TCT-motif	1	part of a light responsive element
TGA-element	1	auxin-responsive element
TGACG-motif	1	*cis*-acting regulatory element involved in the MeJA-responsiveness

**Figure 4:**
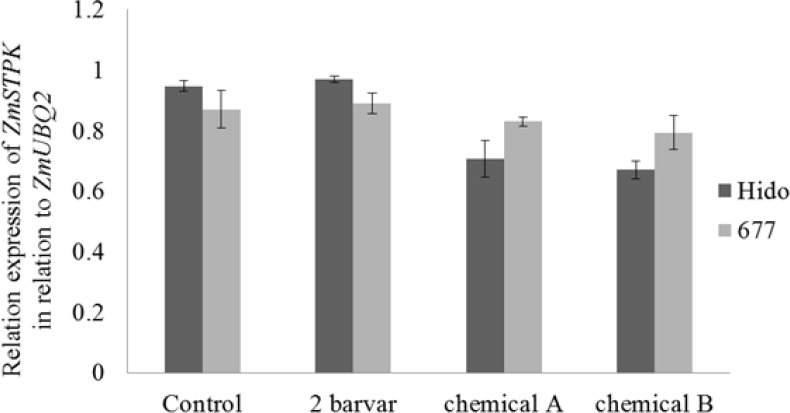
Semiquantitative analysis of the expression level of *ZmSTPK1* gene in the leaves of two maize cultivars, treated with different fertilizers. Column with the same letters are not significantly different at the 5% level using Duncan's multiple range test

The expression of *ZmSTPK1* normalized with *ZmUBQ2* as reference gene. The results indicated that the expression of *ZmSTPK1* increased in control plants and those treated with Barvar-2, while the expression of this gene did not increase under chemical fertilizer 100 and 200 kg/ha, (Fig. 4). 

The statistical analysis revealed no difference for *ZmSTPK1* expression between different levels of triple super phosphate. It can be concluded that some *cis* elements like P1SB, in the promoter region of *ZmSTPK1*, which may be the major factor in phosphate starvation stress response. It is consistent with that of Li et al. (2015) who showed *P1BS* is an important *cis* element in the phosphate signaling pathway. So, the expression of *ZmSTPK1* responded to available phosphate, and its expression up-regulated in control plants and the plants were inoculated with barvar2 before planting, because no external phosphate was added. But, the expression of *ZmSTPK1* did not increase under chemical fertilizers and it appears there was no phosphate starvation even under 100 kg/ha triple super phosphate. In addition, the expression pattern of *ZmSTPK1* was studied in two cultivars to remove genetic background effect. The results showed that its expression pattern was the same in both cultivars, which means the expression of *ZmSTPK1* under different phosphate fertilizer application is independent from genetic background. 
